# Alkaline sphingomyelinase (ENPP7) attenuates DSS-induced colitis by modulating FOXO1-mediated antioxidative stress responses

**DOI:** 10.1186/s12876-026-04832-3

**Published:** 2026-04-14

**Authors:** Wenting Cao, Yuqi Cheng, Xu Wang, Lingqi Wang, Rui Li, Siting Pei, Guihua Zhang, Jindong Ding Petersen, Ping Zhang

**Affiliations:** 1 Key Laboratory of Tropical Translational Medicine of Ministry of Education, School of Public Health, Hainan Academy of Medical Sciences, Hainan Medical University, No.3 Xueyuan Road, Haikou, Hainan Province 571199 China; 2 Department of Clinical Laboratory, Anshan Hospital, First Affiliated Hospital of China Medical University, Anshan, Liaoning Province 114039 China; 3 Department of Laboratory Diagnosis, Qiqihar Tuberculosis Control Center, Qiqihar, Heilongjiang Province 161000 China; 4 Department of Laboratory Diagnosis, The Fifth Affiliated Hospital of Harbin Medical University, Daqing, Heilongjiang Province 163319 China; 5 Department of Public Health, University of Copenhagen, Copenhagen, 1353 Denmark; 6 Department of Public Health, University of Southern Denmark, Odense, 5000 Denmark

**Keywords:** Alkaline sphingomyelinase (ENPP7), FOXO1, Antioxidative stress, DSS-induced colitis

## Abstract

**Background:**

Alkaline sphingomyelinase (alk-SMase), also known as ectonucleotide pyrophosphatase/phosphodiesterase 7 (ENPP7), is an intestinal enzyme involved in sphingolipid metabolism and has been implicated in the regulation of inflammation. However, its role in intestinal inflammation and the underlying mechanisms remain unclear. This study aimed to investigate the role of ENPP7 in dextran sulfate sodium (DSS)-induced colitis, with a particular focus on oxidative stress and FOXO1-related signaling pathways.

**Methods:**

ENPP7 knockout (KO) and wild-type (WT) mice were used to establish a DSS-induced colitis model. Disease severity was assessed by body weight change, disease activity index (DAI), colon length, histopathological analysis, and plasma oxidative stress markers. Levels of pro-inflammatory cytokines and antioxidant enzymes were measured using standard biochemical assays. In vitro, polarized Caco-2 cells were subjected to ENPP7 knockdown and FOXO1 overexpression to evaluate their roles in antioxidative responses.

**Results:**

ENPP7 deficiency significantly aggravated DSS-induced colitis, as evidenced by greater body weight loss, higher DAI scores, and shorter colon length. This effect was accompanied by reduced FOXO1 expression, and was associated with diminished antioxidant defense and mitochondrial dysfunction-related alterations. Additionally, KO mice showed increased levels of pro-inflammatory cytokines (IL-1β and TNF-α) and decreased activities of antioxidant enzymes (CAT and SOD1) in intestinal mucosal tissues compared with WT mice. In Caco-2 cells, ENPP7 knockdown reduced FOXO1 expression, which was associated with impaired antioxidant capacity, whereas FOXO1 overexpression partially reversed these effects.

**Conclusions:**

ENPP7 attenuates DSS-induced colitis, at least in part, by modulating FOXO1-mediated antioxidant responses, thereby influencing oxidative stress and inflammatory processes. These findings highlight ENPP7 as a potential therapeutic target for ulcerative colitis, although further mechanistic and clinical studies are warranted.

**Supplementary Information:**

The online version contains supplementary material available at 10.1186/s12876-026-04832-3.

## Introduction

Ulcerative colitis (UC), a form of inflammatory bowel disease (IBD), is a chronic condition characterized by recurrent episodes of inflammation in the colon, increasing the risk of colorectal cancer [1–3]. Despite extensive research, the exact pathogenesis of UC remains incompletely understood. Oxidative stress plays a central role in UC pathogenesis by disrupting intestinal homeostasis and damaging epithelial barrier integrity [4]. When the intestinal mucosa is exposed to inflammatory agents, including pathogens and reactive oxygen species (ROS), cells exhibit an elevated antioxidant response [5, 6]. While antioxidant responses attempt to counteract reactive oxygen species (ROS), excessive oxidative stress overwhelms cellular defense mechanisms, leading to inflammation and mucosal injury [7]. This results in damage to the barrier function of the intestine and increased intestinal permeability, ultimately worsening the disease condition [8]. This oxidative damage triggers a vicious cycle of inflammation and tissue injury, perpetuating the chronic nature of UC. Understanding the precise mechanisms by which oxidative stress influences the inflammatory process in UC is critical for developing targeted therapeutic strategies. Current research efforts are focused on identifying key molecular pathways involved in the oxidative stress response and exploring potential interventions that can enhance antioxidant defenses in the intestinal mucosa [9].

Alkaline sphingomyelinase (alk-SMase), also known as Ectonucleotide Pyrophosphatase/Phosphodiesterase 7 (ENPP7), is a key enzyme in the gut that hydrolyzes phospholipid molecules, including sphingomyelin (SM), lysophosphatidylcholine (lyso-PC), and platelet-activating factor (PAF). It plays a potential protective role against intestinal inflammation [10, 11]. Recent studies suggest that ENPP7 plays a protective role against colitis by modulating intestinal barrier integrity and enhancing antioxidative defense through transcriptional regulation of the Nrf2 signaling pathway [12]. In addition, Forkhead box O (FoxO) transcription factors are key regulators of cellular homeostasis, particularly in response to oxidative stress [13]. Among them, FOXO1 plays an important role in maintaining redox balance by regulating the expression of antioxidant enzymes such as superoxide dismutase (SOD) and glutathione peroxidase (GSH-Px) [14–17]. Emerging evidence suggests that FOXO1 is involved in the pathogenesis of inflammatory bowel disease (IBD), where its dysregulation is associated with increased oxidative stress and immune imbalance [18, 19]. However, the antioxidant mechanisms in the intestinal mucosa are highly complex, and whether ENPP7 is involved in the regulation of FOXO1-mediated oxidative stress in colitis remains unclear and warrants further investigation.

This study investigates how ENPP7 alleviates intestinal inflammation by regulating FOXO1 and antioxidant enzymes. We aimed to explore the mechanisms by which ENPP7 regulates these antioxidant enzymes to reduce oxidative stress and inflammation in the intestinal epithelium. Our findings could provide insight into novel therapeutic targets for UC.

## Materials and methods

### Establishment of the experimental animal and colitis model

ENPP7 knockout (KO) mice on a C57BL/6 background were generated using Cre/LoxP-mediated gene recombination and embryonic stem cell technology. These mice were generously provided by Prof. Duan (Lund University, Sweden). Wild-type (WT) and ENPP7 KO mice used in this study were obtained by intercrossing heterozygous *ENPP7*^+*/−*^ mice, and genotypes were confirmed by PCR. Mice aged 12 weeks were housed in a specific pathogen-free (SPF) facility at Hainan Medical University under controlled environmental conditions, with ad libitum access to standard chow and water.

To induce experimental ulcerative colitis, all mice (WT and KO) were administered 2.5% (w/v) dextran sulfate sodium (DSS, MW 36,000–50,000; MP Biomedicals, USA) in drinking water ad libitum for 6 consecutive days, while control animals were provided with sterilized tap water. Disease activity index (DAI) scores were recorded daily based on body weight loss, stool consistency, and presence of fecal blood, following established scoring criteria [12].

At the end of the experimental period, all mice were anesthetized using inhalation of isoflurane (RWD Life Science, China) until complete loss of pedal reflex was observed. While under deep anesthesia and with no signs of pain perception, venous blood was collected via orbital sinus puncture. Mice were then humanely euthanized by cervical dislocation. All procedures were approved by the Institutional Animal Care and Use Committee of Hainan Medical University (Approval No. HYLL-2024–275).

### Examination of gross changes in tissues

After 6 days of DSS treatment, the plasma, liver, spleen, thymus, and colon of all the mice were collected for analysis. Surgical procedures were performed under isoflurane anesthesia to ensure a painless and humane process. The colon was removed, and its length was measured from the ileocecal valve to the end of the rectum. The segments were cut open, and the gross changes in the tissue, including the degrees of edema, bleeding, and ulceration, were examined under a dissecting microscope and scored [10]. The liver, spleen, and thymus were removed, and their wet weights were determined.

### Transmission electron microscopy analysis

Colonic tissues (0.5 cm) were immediately fixed in 2% glutaraldehyde at 4 °C for 24 h. After primary fixation, the tissues were postfixed, dehydrated, and embedded. Ultrathin Sects. (60 nm) were cut and stained with 1% uranyl acetate for 20 min and lead citrate for 7 min to enhance contrast. The ultrastructural morphology of the colonic epithelial cells was examined using transmission electron microscopy. Images were captured digitally and analyzed to assess the integrity of cellular organelles, including microvilli, mitochondria, and the overall cellular architecture.

### Examination of plasma antioxidant levels

The levels of malondialdehyde (MDA) and the activities of total SOD (T-SOD) and glutathione peroxidase (GSH-Px) in plasma were assessed using kits from Nanjing Jiancheng Bioengineering Institute (Nanjing, China) following the manufacturer’s protocols. SOD activity was evaluated by its capacity to inhibit the oxidation of hydroxylamine by superoxide radicals generated from the xanthine-xanthine oxidase reaction. One unit of SOD activity was defined as the quantity that caused a 50% reduction in absorbance at 550 nm. GSH-Px activity was determined by measuring the rate at which it catalyzed the oxidation of reduced glutathione to oxidized glutathione in the presence of hydrogen peroxide. MDA concentrations were determined using the thiobarbituric acid (TBA) method. This technique involves spectrophotometric analysis of the color change that occurs when TBA reacts with MDA. The absorbance of the TBA-MDA complex was measured at 532 nm to calculate the MDA levels.

### Culture and treatment of Caco-2 cells

Caco-2 cells were purchased from the American Type Culture Collection and cultured in DMEM supplemented with 15% fetal bovine serum (FBS), 1% penicillin, streptomycin, nonessential amino acids, glutamine, and sodium pyruvate in a humidified incubator with 5% CO_2_ at 37 °C. In this study, Caco-2 cells were polarized by culture on Transwell plates for 21 days [20]. The differentiated Caco-2 cell model was then used for subsequent transfection experiments. Caco-2 cells were treated with 1% DSS for 12 h to mimic inflammatory conditions [21].

### siRNAs and plasmid design and transfection

Small interfering RNAs (siRNAs) targeting the *ENPP7* gene were designed and synthesized by GenePharma (Suzhou, China), with nontargeted control siRNA (NC) used as a negative control. The FOXO1 overexpression plasmid was constructed by GenePharma (Suzhou, China), with the empty vector used as a negative control (NC). Transfection was performed according to the instructions provided with the Lipofectamine 2000 Reagent (Thermo Fisher Scientific, Shanghai, China). Briefly, 8 μL of 10 μM siRNA solution or 3 μg plasmid DNA was diluted in 250 μL serum-free DMEM, and 8 μL Lipofectamine 2000 was diluted separately in 250 μL serum-free DMEM. After incubation for 5 min, the solutions were combined and incubated for an additional 20 min at room temperature. The resulting 500 μL transfection complex was added to the apical chamber of each 6-well Transwell insert containing 1 mL serum-free medium. After 6 h of incubation, the medium was replaced with fresh complete medium, and the cells were further cultured for 24 h.

### RNA extraction and RT‒qPCR

RNA was extracted from cells and colon tissue using TRIzol (Thermo Fisher Scientific, Shanghai, China) according to the manufacturer's protocol. For colon tissues and Caco-2 cells treated with DSS, lithium chloride was added to remove the inhibitory effect of DSS on qPCR, and the samples were allowed to stand at 4 °C for 2 h, followed by centrifugation and repetition [22]. The isolated RNA was reverse transcribed into cDNA using a qPCR RT Master Mix Kit (Toyobo, Osaka, Japan). The cDNA samples were used to measure the mRNA expression levels using a SYBR Green Real-time PCR Master Mix Kit (Toyobo, Osaka, Japan). GAPDH was used as an internal control for mRNA quantification. Threshold cycle (Ct) values were determined, and relative mRNA levels were normalized to GAPDH and calculated using the 2^−ΔΔCt^ method, with the WT group before DSS treatment as the calibrator for normalization across experiments. All samples were analyzed in triplicate and the average Ct values were used for analysis. The primers used were as follows:

**Table:** 

Gene	Species	Primer Direction	Sequence (5' to 3')
*Enpp7*	Mouse	Forward	CTGCCACTTTACACTGGTCAC
*Enpp7*	Mouse	Reverse	TGGCACTGAGGCGAGAAC
*GAPDH*	Mouse	Forward	GGTTGTCTCCTGCGACTTCA
*GAPDH*	Mouse	Reverse	TGGTCCAGGGTTTCTTACTCC
*IL-1β*	Mouse	Forward	CACTACAGGCTCCGAGATGAACAAC
*IL-1β*	Mouse	Reverse	TGTCGTTGCTTGGTTCTCCTTGTAC
*IL-4*	Mouse	Forward	TACCAGGAGCCATATCCACGGATG
*IL-4*	Mouse	Reverse	TGTGGTGTTCTTCGTTGCTGTGAG
*IL-10*	Mouse	Forward	AGAGAAGCATGGCCCAGAAATCAAG
*IL-10*	Mouse	Reverse	CTTCACCTGCTCCACTGCCTTG
*Foxo1*	Mouse	Forward	ACATCTGCCATGAACCGCTTGAC
*Foxo1*	Mouse	Reverse	CACCCATCCTACCATAGCCATTGC
*Cat*	Mouse	Forward	GGAGGCGGGAACCCAATAGGAG
*Cat*	Mouse	Reverse	TGTCAAAGTGTGCCATCTCGTCAG
*Sod1*	Mouse	Forward	TCCCAGACCTGCCTTACGACTATG
*Sod1*	Mouse	Reverse	CTCCTCGGTGGCGTTGAGATTG
*Gpx2*	Mouse	Forward	AGGGCTGTGCTGATTGAGAATGTG
*Gpx2*	Mouse	Reverse	CTCCTGATGTCCGAACTGGTTGC
*ENPP7*	Human	Forward	CCGGAAAGAAGGCATCGCAC
*ENPP7*	Human	Reverse	CTCCTCTGTGAACCACGCCA
*GAPDH*	Human	Forward	CAGGAGCATTGCTGATGAT
*GAPDH*	Human	Reverse	GAAGGCTGGGGCTCATTT
*IL-1β*	Human	Forward	GGACAGGATATGGAGCAACAAGTGG
*IL-1β*	Human	Reverse	TCATCTTTCAACACGCAGGACAGG
*FOXO1*	Human	Forward	CCTGGACATGCTCAGCAGACATC
*FOXO1*	Human	Reverse	CACTTGGGTCAGGCGGTTCATAC
*CAT*	Human	Forward	ATGCAGGACAATCAGGGTGG
*CAT*	Human	Reverse	TTGAATCTCCGCACTTCTCCA
*SOD1*	Human	Forward	GATGACTTGGGCAAAGGTGGAAATG
*SOD1*	Human	Reverse	CCAATTACACCACAAGCCAAACGAC
*GPX2*	Human	Forward	CTTCTATGACCTCAGTGCCATCAGC
*GPX2*	Human	Reverse	AGAGCGAAGCCACATTCTCAATCAG

### Western blot analysis

Colon tissue and Caco-2 cells were lysed by sonication on ice with lysis buffer (50 mM Tris, pH 7.4, 150 mM NaCl, 1% Triton X-100, 1 mM EDTA, and 2 mM PMSF), followed by centrifugation at 13,500 rpm for 20 min at 4 °C to collect the supernatants. Protein samples (20 μg) were separated on 8% SDS‒PAGE gels and transferred onto nitrocellulose membranes. After blocking with 5% nonfat milk for 1.5 h, the membranes were incubated overnight at 4 °C with primary antibodies against ENPP7 (1:250) (Bio-Techne, Minneapolis, USA), FOXO1 (1:1000) (Abcam, Cambridge, United Kingdom), SOD1 (1:1000) (Proteintech, Rosemont, USA), CAT (1:2000) (Proteintech), IL-1β (1:1000) (Proteintech), and β-actin (1:4000). The membranes were then incubated with horseradish peroxidase-conjugated secondary antibodies. Protein detection was performed using Super ECL reagent (HaiGene, M2301). Protein bands were visualized using enhanced chemiluminescence (ECL) and recorded by X-ray film exposure in a darkroom.

### Tissue immunofluorescence

Fresh colon tissues were fixed in 4% paraformaldehyde for 2 days, dehydrated, embedded in paraffin, and sectioned into 5 μm thick slices. The sections were subjected to antigen retrieval by heating in a sodium citrate solution in a microwave oven for 15 min, followed by cooling to room temperature. The sections were then blocked with 3% H_2_O_2_ in PBS at room temperature to quench endogenous peroxidase activity and incubated overnight at 4 °C with primary antibodies against FOXO1 (1:100), SOD1 (1:50), and CAT (1:50). Following primary antibody incubation, the samples were washed three times with PBS and then incubated with fluorescently labeled secondary antibodies (1:50) diluted in PBS at 37 °C for 2 h. Finally, DAPI (1:10) was applied for 15 min at room temperature to counterstain the nuclei. Images were acquired using a confocal laser scanning microscope, enabling detailed visualization of protein localization and expression.

### Cell immunofluorescence

Caco-2 cells were cultured on sterile glass coverslips under normal or drug administration conditions for 12 h after transfection in 24-well plates. The cells were fixed in 4% paraformaldehyde for 15 min, permeabilized with 0.3% Triton X-100 for 30 min, and blocked with 3% normal goat serum for 1 h at 37 °C. The samples were then incubated overnight at 4 °C with primary antibodies against FOXO1 (1:100), SOD1 (1:50), CAT (1:50), and ENPP7 (1:50) (Sino Biological, Beijing, China). After three washes with PBS, the samples were incubated with fluorescently labeled secondary antibodies (1:50) diluted in PBS at 37 °C for 2 h. Finally, DAPI (1:10) was applied for 15 min at room temperature to counterstain the nuclei. Images were acquired using a confocal laser scanning microscope.

### Statistical analysis

Statistical analyses were performed using IBM SPSS Statistics version 27.0 (IBM Corp., Armonk, NY, USA) and GraphPad Prism 8 (GraphPad Software, La Jolla, CA, USA). Data are presented as mean ± SEM. Comparisons between two groups were performed using Student’s t-test. For multiple group comparisons, one-way analysis of variance (ANOVA) followed by Tukey’s multiple comparisons test was applied. A p value < 0.05 was considered statistically significant.

## Results

### ENPP7 deficiency exacerbates disease severity in DSS-induced colitis

**Fig. 1:**
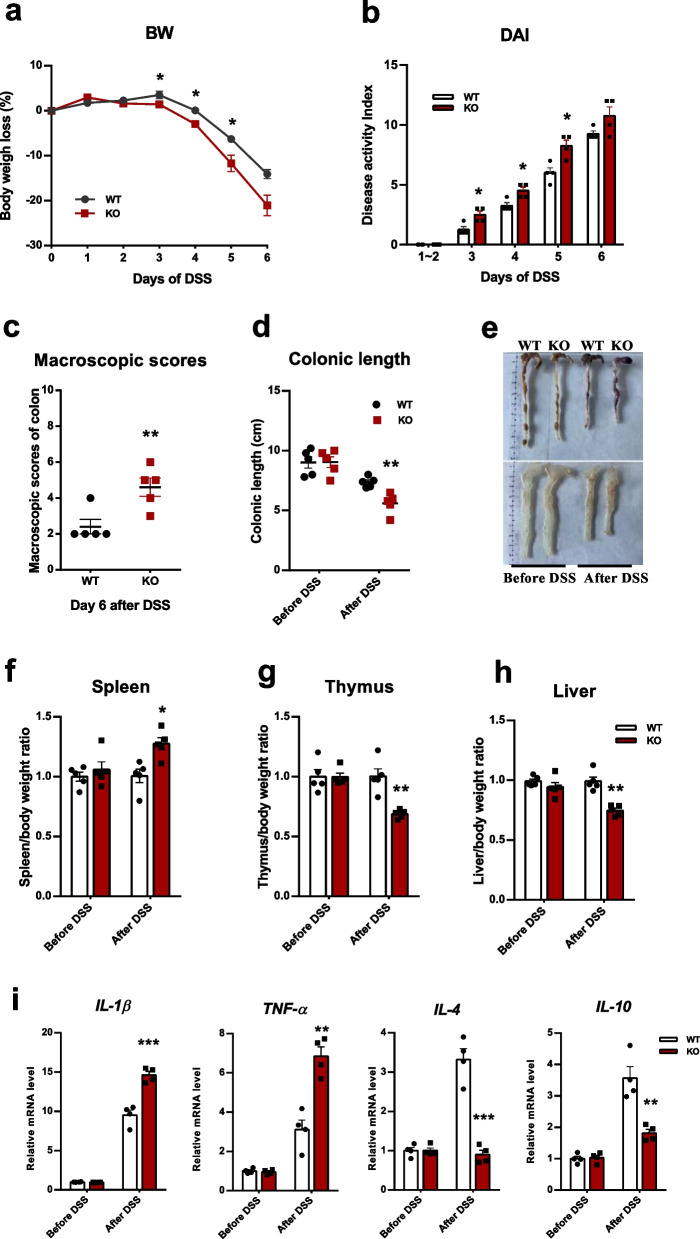
ENPP7 deficiency exacerbates inflammatory responses and disease severity in DSS-induced colitis. All mice (WT and ENPP7-deficient, KO) were administered 2.5% (w/v) DSS in drinking water ad libitum for 6 days to induce colitis. **a** Body weight change over time. **b** Disease Activity Index (DAI) scores during DSS treatment. **c** Colonic length before and after DSS treatment. **d** Macroscopic scores of colons on day 6. **e** Representative images of colons. **f–h** Organ-to-body weight ratios (spleen, thymus, liver). **i** mRNA expression levels of inflammatory cytokines (*IL-1β*, *TNF-α*, *IL-4*, *IL-10*) in colonic tissues. Data are presented as mean ± SEM. For most analyses, *n =* 5 mice per group. For qPCR analysis, *n =* 4 mice per group (biological replicates), and each sample was analyzed in three technical replicates. Statistical analysis was performed using Student’s t-test. WT mice served as controls for KO mice, *P < 0.05, **P < 0.01, ***P < 0.001

In the DSS-induced colitis model, ENPP7-deficient mice exhibited more severe inflammation than WT mice, as evidenced by greater body weight loss, higher DAI scores, shorter colonic length, and increased macroscopic injury. Body weight began to decline from day 3, reaching a loss of 14.09% in WT mice and 21.07% in KO mice by day 6 (Fig. 1a). Consistently, KO mice showed significantly higher DAI scores throughout DSS treatment (Fig. 1b) and markedly shorter colons after 6 days (Fig. 1c), along with more severe macroscopic damage (Fig. 1d, e). In addition, changes in organ indices, including splenomegaly, thymic atrophy, and reduced liver weight, were more pronounced in KO mice (Fig. 1f-h).

To further assess the inflammatory response in colonic mucosal tissues, we examined the expression of representative cytokines. After 6 days of DSS treatment, the expression of the proinflammatory cytokines IL-1β and TNF-α was significantly increased in KO mice compared with WT mice, whereas the anti-inflammatory cytokines IL-4 and IL-10 were significantly decreased (Fig. 1i). These findings further support that ENPP7 deficiency aggravates inflammatory imbalance in the colonic mucosa, consistent with our previous observations [12].

### ENPP7 deficiency aggravates oxidative stress in DSS-induced colitis

**Fig. 2:**
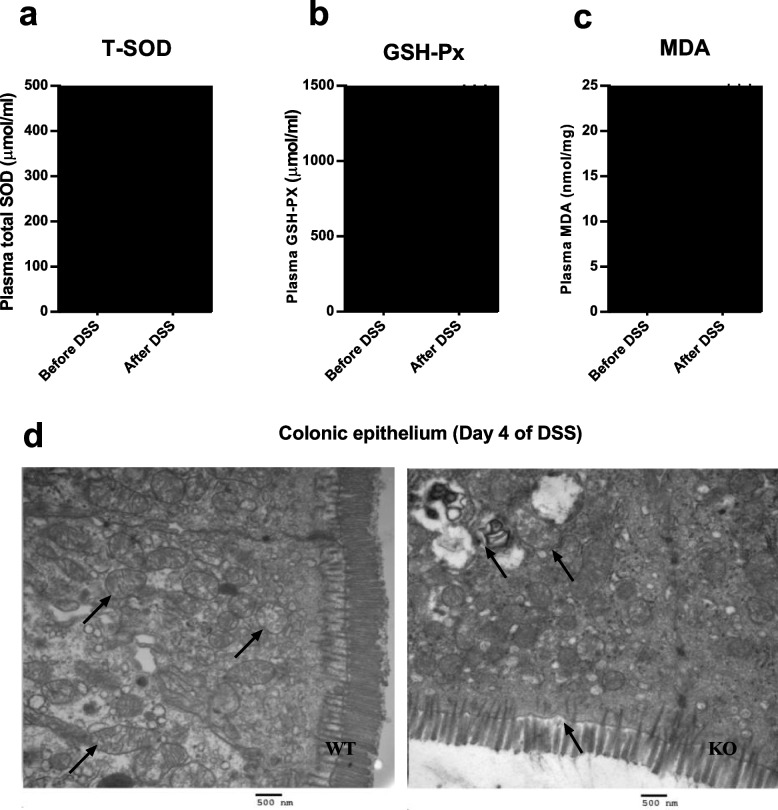
ENPP7 deficiency aggravates oxidative stress in DSS-induced colitis. Plasma and colonic tissues were collected following DSS treatment. **a**-**c** Plasma levels of T-SOD, GSH-Px, and MDA measured on day 6 after DSS treatment. Data are presented as mean ± SEM (*n =* 5 mice per group). Statistical analysis was performed using Student’s t-test. Compared with WT mice, ***P < 0.001. **d** Representative transmission electron microscopy images of colonic epithelial cells obtained on day 4 after DSS treatment (arrows indicate mitochondrial swelling, cristae loss, and vacuolar degeneration)

To assess the antioxidant function of ENPP7 in colitis, we measured the levels of the antioxidant enzymes GSH-Px and SOD in the plasma of mice both before and after DSS treatment. Compared with WT mice, KO mice with DSS-induced colitis exhibited significantly lower antioxidant capacity (Fig. 2a, b). Additionally, the oxidative stress marker MDA in plasma was significantly greater in the KO mice than in the WT mice after 6 days of DSS treatment (Fig. 2c).

### ENPP7 deficiency promotes mitochondrial dysfunction in DSS-induced colitis

To evaluate changes in oxidative stress levels, we examined mitochondrial morphology in colonic epithelial cells. Transmission electron microscopy revealed severe mitochondrial swelling, deformation, and cristae loss in colonic epithelial cells following DSS treatment, suggesting oxidative stress-induced organelle damage. However, these mitochondrial alterations, including myeloid-like changes, vacuolar degeneration and detachment of microvilli in intestinal epithelial cells, were markedly more severe in the ENPP7 KO mice, which exhibited extensive damage, as shown by the arrows in Fig. 2d. These findings collectively indicate that ENPP7 deficiency exacerbates colitis, accompanied by impaired antioxidant defense and mitochondrial dysfunction.

### ENPP7 deficiency reduces FOXO1 and downstream antioxidant enzyme expression in DSS-induced colitis

**Fig. 3:**
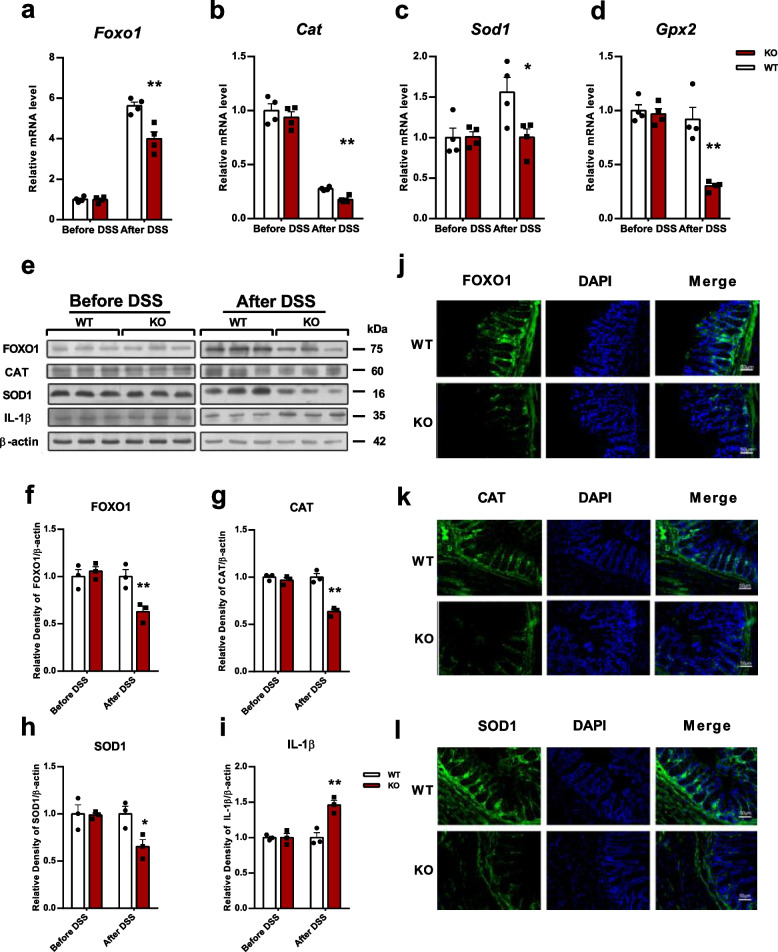
ENPP7 deficiency reduces FOXO1 expression and antioxidant capacity in DSS-induced colitis. Colonic mucosal tissues of all mice were collected after 6 days of DSS treatment. **a**-**d** Relative mRNA expression levels of *Foxo1*, *Cat*, *Sod1*, and *Gpx2* in colonic tissues (*n =* 4 mice per group). **e**-**i** Protein expression levels of FOXO1, CAT, SOD1, and IL-1β in colonic tissues were analyzed by Western blot, with β-actin used as a loading control (*n =* 3 mice per group). **j**-**l** Immunofluorescence staining of FOXO1, CAT, and SOD1 in colonic tissues (DAPI for nuclear staining). Data are presented as mean ± SEM. Statistical analysis was performed using Student’s t-test. Compared with WT mice, *P < 0.05, **P < 0.01

To investigate how ENPP7 regulates oxidative stress levels in colitis, we measured the changes in antioxidant enzymes and the upstream regulatory transcription factor FOXO1 using RT-qPCR and Western blot. In the colonic mucosal tissues, the FOXO1 mRNA and protein levels were significantly lower in the KO mice than in the WT mice after DSS-induced inflammation (Fig. 3a and f). This reduction was accompanied by a significant downregulation of the downstream antioxidant enzymes CAT, SOD1 and GSH-Px 2 (GPX2) and the inflammatory cytokine IL-1β (Fig. 3b-d and g-i). Immunofluorescence staining showed reduced FOXO1, CAT, and SOD1 fluorescence intensity in colonic mucosal tissues after DSS treatment, with a greater reduction in KO mice. Although FOXO1 signal partially overlapped with nuclear staining, its subcellular redistribution was not quantitatively assessed in the present study (Fig. 3j-l).

### ENPP7 knockdown attenuates FOXO1-mediated antioxidant responses in Caco-2 cells

**Fig. 4:**
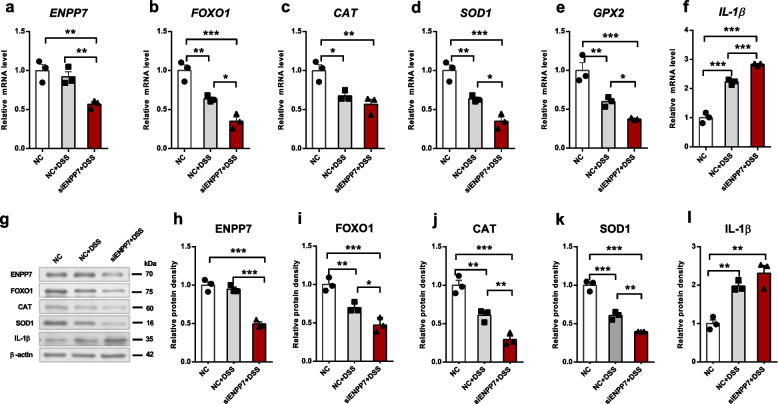
ENPP7 knockdown reduced FOXO1 expression and antioxidant capacity in Caco-2 cells. Polarized Caco-2 cells were transfected with siRNA targeting ENPP7 for 24 h, followed by treatment with 1% DSS for 12 h. The experimental groups were as follows: NC, NC + DSS, and siENPP7 + DSS. **a**-**f** mRNA expression levels of *ENPP7*, *FOXO1*, *CAT*, *SOD1*, *GPX2*, and *IL-1β*. Data are presented as mean ± SEM from three independent experiments, each performed with three technical replicates. **g**-**l** Protein expression levels determined by Western blot. Data are presented as mean ± SEM from three independent experiments, each performed in triplicate. Statistical analysis was performed using one-way ANOVA followed by Tukey’s multiple comparisons test. *P < 0.05, **P < 0.01, ***P < 0.001

To observe how ENPP7 regulates FOXO1-mediated oxidative stress in cells, we knocked down the *ENPP7* gene in Caco-2 cells using siENPP7 and then induced an inflammatory model with 1% DSS. In the cell inflammation model, DSS treatment and the lack of ENPP7 activity led to a significant decrease in FOXO1 expression, indicating that ENPP7 regulates FOXO1. Additionally, the mRNA and protein levels of the downstream proteins CAT, SOD1, and GPX2, which are affected by FOXO1, were also significantly reduced following *ENPP7* knockdown. We observed a significant elevation in the inflammatory cytokine IL-1β in *ENPP7*-knockdown cells, particularly under conditions of severe inflammation (Fig. 4).

### FOXO1 overexpression rescues antioxidant capacity impaired by ENPP7 knockdown in Caco-2 cells

**Fig. 5:**
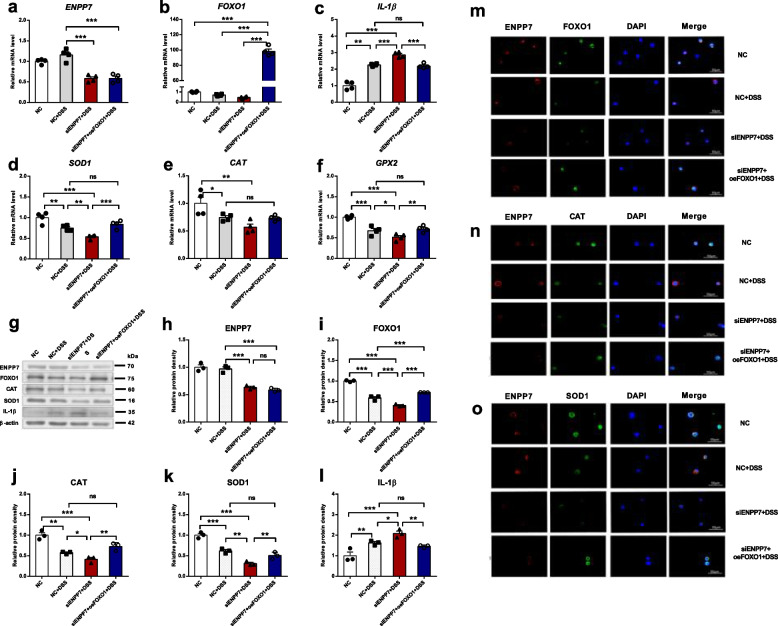
FOXO1 overexpression rescues antioxidant impairment induced by ENPP7 knockdown in Caco-2 cells. Polarized Caco-2 cells were transfected with siENPP7 alone or co-transfected with siENPP7 and a FOXO1 overexpression plasmid for 24 h, followed by treatment with 1% DSS for 12 h. The experimental groups were as follows: NC, NC + DSS, siENPP7 + DSS, and siENPP7 + oeFOXO1 + DSS. **a**-**f** mRNA expression levels. Data are presented as mean ± SEM from four independent experiments, each performed with three technical replicates. **g**-**l** Protein expression levels determined by Western blot. Data are presented as mean ± SEM from three independent experiments, each performed with three technical replicates. **m**–**o** Immunofluorescence staining of ENPP7, FOXO1, CAT, and SOD1 (DAPI was used for nuclear staining). Statistical analysis was performed using one-way ANOVA followed by Tukey’s multiple comparisons test. *P < 0.05, **P < 0.01, ***P < 0.001; ns, not significant

To demonstrate that the antioxidant enzymes CAT, SOD1, and GPX2 are regulated by FOXO1, we performed a rescue experiment by overexpressing FOXO1 in cells with reduced FOXO1 expression due to *ENPP7* knockdown. After FOXO1 overexpression, we again observed changes in antioxidant enzymes. In DSS-induced inflammatory cells with FOXO1 overexpression, the mRNA and protein levels of antioxidant enzymes were significantly increased, restoring them to levels comparable to those observed with ENPP7 expression. We further examined the localization and fluorescence intensity of FOXO1, CAT, and SOD1 in cells using fluorescence staining. ENPP7 was primarily expressed on the cell membrane, while FOXO1 expression overlapped with that in the cell nucleus, and antioxidant enzymes were expressed in the cytoplasm. The fluorescence intensity, indicating the level of expression, was consistent with the results of previous experiments (as shown in Fig. 5).

## Discussion

In this study, we demonstrate for the first time that ENPP7 deficiency aggravates DSS-induced colitis, which is associated with impaired antioxidant capacity and mitochondrial dysfunction. These findings support a protective role of ENPP7 in intestinal inflammation. Previous studies have reported that intestinal ENPP7 activity is significantly decreased in patients with chronic ulcerative colitis [23]. Supplementation with recombinant ENPP7 effectively alleviated ulcerative colitis in rats, indicating the anti-inflammatory role of ENPP7 in intestinal inflammation [24]. Recently, our research revealed that ENPP7 can alleviate intestinal inflammation through its antioxidant effects in DSS-induced colitis [12]. However, the precise underlying mechanisms remain unclear. To investigate the anti-inflammatory and antioxidative roles of ENPP7, we established a 2.5% DSS-induced colitis model in ENPP7 KO and WT mice, which models aspects of human ulcerative colitis and enables the assessment of early inflammatory and oxidative changes [25].

After 6 days of DSS induction, ENPP7-deficient mice exhibited significantly more severe inflammatory responses than did WT mice, as evidenced by a marked decrease in body weight and a significant increase in DAI. Further assessments of colon length and colon macroscopic scores also revealed similar trends, consistent with our previous studies, thus validating the reliability of the DSS-induced colitis model used in this study [10, 12]. Further investigation of the organ-to-body weight ratios of immune-related organs revealed that ENPP7 deficiency led to more splenomegaly in DSS-induced colitis mice, along with significant atrophy of the thymus and liver, indicating more severe systemic immune damage [26]. These results corroborate the protective role of ENPP7 activity in colitis against DSS-induced inflammation.

Subsequent analysis of cytokine levels in intestinal mucosal tissues revealed that, after DSS induction, ENPP7 KO mice exhibited significantly elevated levels of the proinflammatory cytokines IL-1β and TNF-α, along with reduced levels of the anti-inflammatory cytokines IL-4 and IL-10 compared with WT mice [27–29]. These findings suggest that ENPP7 deficiency disrupts the balance between pro- and anti-inflammatory responses in the colonic mucosa. ENPP7 is known to hydrolyze sphingomyelin to generate ceramide, and ceramide metabolism has been reported to be associated with IL-10 regulation in the intestine [30]. Therefore, ENPP7 deficiency may alter ceramide production, which could contribute to the reduced IL-10 levels observed in this study. In subsequent in vitro experiments, IL-1β was selected as a representative marker to evaluate inflammatory responses. Overall, these cytokine alterations support a role for ENPP7 in maintaining immune homeostasis in the colonic mucosa.

To explore whether the anti-inflammatory effects of ENPP7 are associated with oxidative stress regulation, we measured the plasma levels of the oxidative stress marker MDA and the key antioxidant enzymes GSH-Px and SOD1, which play essential roles in scavenging reactive oxygen species and maintaining redox homeostasis. Under physiological conditions, the oxidative and antioxidant systems are in dynamic equilibrium [31]. ENPP7 deficiency was associated with reduced antioxidant enzyme levels in colitis, indicating that ENPP7 plays a critical role in regulating oxidative stress. Therefore, we considered that ENPP7 deficiency might lead to heightened inflammatory responses and immune damage in DSS-induced colitis, partly through the modulation of antioxidative stress pathways.

In the present study, transmission electron microscopy was performed at 4 days after DSS induction to capture early ultrastructural changes before the onset of severe tissue damage. This time point was selected based on the clinical status of the mice, disease activity index scores, and previous literature, as it represents an early stage of colitis characterized by subtle cellular and subcellular alterations [25]. Mitochondria are the primary site of ROS production, and ROS are crucial for regulating transcription factors that control the expression of antioxidant enzymes [32, 33]. Ultrastructural analysis revealed evident mitochondrial damage in intestinal epithelial cells following DSS treatment, including swelling, degeneration, and loss of cristae. These alterations were more pronounced in ENPP7-deficient mice, suggesting that ENPP7 plays a protective role in maintaining mitochondrial integrity under inflammatory conditions [7]. Given that mitochondria are a major source of ROS, such structural damage may reflect disrupted redox homeostasis and contribute to oxidative stress. Collectively, these findings indicate that ENPP7 may mitigate early mitochondrial dysfunction and oxidative stress during colitis development.

Given the close interplay between mitochondrial function, ROS production, and antioxidant signaling pathways, we next investigated whether FOXO1 is involved in ENPP7-mediated redox regulation. FOXO1 has been recognized as a key regulator of oxidative stress and is known to control the expression of multiple antioxidant enzymes [17]. In the present study, following 6 days of DSS induction, ENPP7-deficient mice exhibited significantly reduced FOXO1 expression and decreased levels of antioxidant enzymes in the colonic mucosa compared with WT mice. In parallel, antioxidant enzyme activities in plasma were also reduced, while MDA levels were increased, indicating impaired systemic antioxidant capacity and enhanced oxidative stress. However, we did not assess FOXO1 expression or antioxidant enzyme levels at the early (4-day) time point. Therefore, it remains unclear whether downregulation of the FOXO1 pathway precedes overt tissue injury or occurs as a consequence of inflammatory damage. Taken together, these findings suggest that ENPP7 may, at least in part, exert its antioxidant effects through modulation of FOXO1.

Notably, given that ENPP7 is an extracellular enzyme primarily involved in sphingolipid metabolism [34], it is more likely to regulate FOXO1 indirectly rather than through a direct interaction. One possible explanation is that ENPP7 deficiency alters the intestinal microenvironment, leading to enhanced oxidative stress or mitochondrial dysfunction, which in turn suppresses FOXO1 activity. Although the precise upstream mechanisms remain to be elucidated, our data support the antioxidant effects of ENPP7 mediated by FOXO1 in intestinal inflammation. Collectively, these findings highlight a potential protective role of ENPP7 in maintaining redox homeostasis and alleviating oxidative stress-associated intestinal inflammation.

Building upon the above findings suggesting that ENPP7 may indirectly regulate FOXO1 through lipid-mediated signaling pathways, we further explored this mechanism using an in vitro model of intestinal epithelial cells. To this end, Caco-2 cells were cultured on Transwell inserts for 21 days to achieve polarization and mimic the intestinal epithelial barrier. Consistent with previous studies [20], ENPP7 activity increased by more than tenfold under polarized conditions, indicating its functional relevance in differentiated intestinal epithelial cells. Therefore, we focused on evaluating the effects of ENPP7 knockdown. Interestingly, ENPP7 expression itself did not change significantly following inflammatory stimulation, which may be attributed to the fact that DSS primarily induces epithelial barrier disruption rather than directly regulating ENPP7 expression. Instead, inflammatory conditions may alter ENPP7 conformation and impair its enzymatic activity independently of expression levels [35]. Notably, ENPP7 knockdown in the presence of inflammatory stimulation led to a marked reduction in FOXO1 expression, supporting a regulatory link between ENPP7 and FOXO1 under stress conditions.

To further investigate whether ENPP7 regulates FOXO1-mediated antioxidant responses during inflammation, we performed a functional rescue experiment by combining ENPP7 knockdown with FOXO1 overexpression. As expected, FOXO1 overexpression partially rescued the downregulation of antioxidant enzymes induced by ENPP7 knockdown. This finding suggests that the decrease in antioxidant capacity caused by ENPP7 deficiency is mediated, at least in part, through suppression of FOXO1 under inflammatory conditions.

Previous studies have shown that FOXO1 acts as a key sensor of intracellular ROS and plays a central role in regulating cellular oxidative stress responses [17]. In the context of ENPP7 deficiency and DSS-induced inflammation, we observed mitochondrial swelling and structural degeneration in colonic tissues, indicating mitochondrial dysfunction. Although intracellular ROS levels were not directly measured in this study, such ultrastructural alterations are widely recognized as hallmarks of oxidative stress and disrupted redox homeostasis, and may indirectly reflect increased ROS accumulation. This oxidative imbalance may impair FOXO1 activity and its ability to maintain antioxidant defense, thereby contributing to reduced cellular antioxidant capacity and exacerbated inflammatory responses. Additionally, our data demonstrate that ENPP7 deficiency is associated with reduced FOXO1 expression and impaired antioxidant capacity. However, the precise mechanisms linking ENPP7 to FOXO1 regulation remain to be fully elucidated. It is therefore plausible that ENPP7 may indirectly modulate FOXO1 activity by influencing intracellular oxidative stress status or upstream kinase signaling pathways, thereby affecting FOXO1 transcriptional activity and downstream antioxidant gene expression. However, these potential mechanisms were not directly investigated in the present study and warrant further exploration.

In addition, studies have shown that FOXO1 is crucial for maintaining intestinal homeostasis by regulating mucus secretion in goblet cells. Loss of FOXO1 in intestinal epithelial cells leads to defects in goblet cell autophagy and mucus secretion, resulting in an impaired gut microenvironment and increased susceptibility to intestinal inflammation [36]. Previous research from our group has indicated that ENPP7 can maintain intestinal homeostasis by protecting the integrity of the gut barrier [12]. Although this study did not explore whether ENPP7 exerts its protective effects on the intestinal barrier through FOXO1-mediated mechanisms, the aforementioned findings suggest that further investigation into the molecular mechanisms by which ENPP7 protects the intestine, potentially involving FOXO1, is warranted.

Taken together, our results support the existence of an ENPP7-FOXO1-antioxidant regulatory axis, in which ENPP7 deficiency suppresses FOXO1 expression, leading to mitochondrial dysfunction, impaired antioxidant defense, and aggravated intestinal inflammation. These findings provide new insights into the role of ENPP7 in regulating redox homeostasis and inflammatory responses in the intestinal mucosa. However, this study has several limitations. Although our findings support a functional link between ENPP7 and FOXO1-mediated antioxidant regulation, the precise molecular mechanisms underlying this interaction remain to be fully elucidated. In particular, the involvement of mitochondrial antioxidant systems and upstream signaling pathways warrants further investigation. Moreover, intracellular ROS levels were not directly assessed in this study; incorporating direct measurements of oxidative stress in future work would strengthen the mechanistic interpretation. Finally, validation in clinical samples from patients with ulcerative colitis is required to confirm the translational relevance of our findings.

## Conclusions

This study highlights the potential role of ENPP7 in reducing oxidative stress and inflammation in DSS-induced colitis, potentially through the regulation of FOXO1 and its downstream antioxidant enzymes. We found that ENPP7 deficiency was associated with increased inflammation and reduced FOXO1 expression, which may contribute to impaired antioxidant defenses and elevated proinflammatory cytokines. These findings suggest that ENPP7 may represent a promising therapeutic target for inflammatory bowel disease, particularly ulcerative colitis. By providing insight into the molecular mechanisms underlying intestinal inflammation and oxidative stress, this study may contribute to the development of novel therapeutic strategies aimed at enhancing mucosal protection and reducing inflammation.

## Supplementary Information

Supplementary Material 1.

## Data Availability

The datasets used and analyzed during the current study are available from the corresponding author upon reasonable request. Unprocessed original Western blot images and other supporting raw data are also available from the corresponding author upon reasonable request.

## Abbreviations

alk-SMase: Alkaline sphingomyelinase
CAT: Catalase
DAI: Disease activity index
DSS: Dextran sulfate sodium
ENPP7: Ectonucleotide Pyrophosphatase/Phosphodiesterase 7
FBS: Fetal bovine serum
FOXO1: Forkhead box transcription factor O1
GPX2: Glutathione peroxidase 2
GSH-Px: Glutathione peroxidase
IBD: Inflammatory bowel disease
lyso-PC: Lysophosphatidylcholine
MDA: Malondialdehyde
PAF: Platelet-activating factor
ROS: Reactive oxygen species
siRNAs: Small interfering RNAs
SM: Sphingomyelin
SOD: Superoxide dismutase
TBA: Thiobarbituric acid
UC: Ulcerative colitis
