# Supplementary material for: Alkaline sphingomyelinase (ENPP7) attenuates DSS-induced colitis by modulating FOXO1-mediated antioxidative stress responses
Source: BMC Gastroenterol. 2026 Apr 14;26:315. doi: 10.1186/s12876-026-04832-3 (PMC13188645; doi:10.1186/s12876-026-04832-3)
Supplement: Supplementary file 1 — Supplementary Material 1. [file 12876_2026_4832_MOESM1_ESM.zip › Western Blot Explanation and Marker Info.pdf]

Submission ID: b63e4f0e-2262-4574-8801-e5176136ea79

Western Blot Image Explanation and Supporting Information

All Western blot membranes in this study were developed using **X-ray film exposure in a darkroom**. Prior to exposure, we manually cut a piece of X-ray film to match the size of each membrane and carefully overlaid it onto the blot for development.

During membrane trimming, the **location of the pre-stained protein marker** was used as a reference to guide cutting. However, since the **marker bands are not chemiluminescent**, they do not appear on the final film images developed using ECL reagents. This is a common and well-known limitation of film-based Western blot detection, which only captures chemiluminescent signals from HRP-conjugated antibodies.

We confirm that the protein ladder was used during electrophoresis and membrane preparation, and molecular weight estimations were properly referenced at the time of exposure.

All Western blot results presented in the manuscript are based on repeated experiments with multiple independent biological samples. The band patterns shown are representative and were consistently reproducible across replicates, ensuring the **accuracy and reliability** of the findings.

Figure 3e Raw Western Blot Image

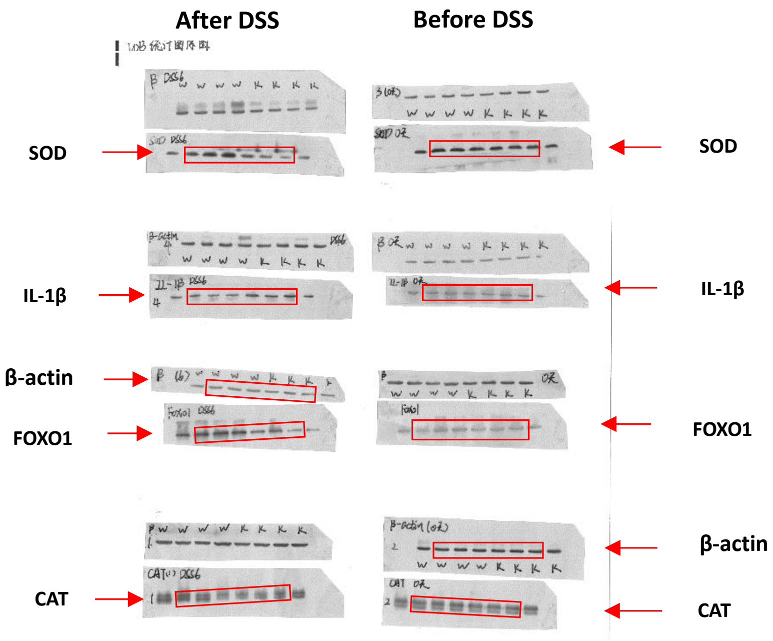

Each Western blot membrane shown in Figure 3e contained **eight sample lanes**, loaded in the following order:

- **Lanes 1–4: WT**
- **Lanes 5–8: KO**

The first four lanes represent four independent samples from the WT group, followed by **four independent samples from the KO group**.

Due to minor electrophoretic distortion at the edges of the membrane, the bands in the **leftmost (Lane 1)** and **rightmost (Lane 8)** positions were excluded from analysis. For the purpose of figure assembly, **the central three WT lanes (Lanes 2–4)** and **three KO lanes (Lanes 5–7)** were selected and presented in the composite figure.

The bands used for **Figure 3e** are clearly indicated in the raw blot by a visible bounding box (red rectangles indicate the specific regions that were cropped and presented in the main manuscript figures). These bands were **cropped from the original membrane without any image manipulation or enhancement**.

Figure 4g Raw Western Blot Image

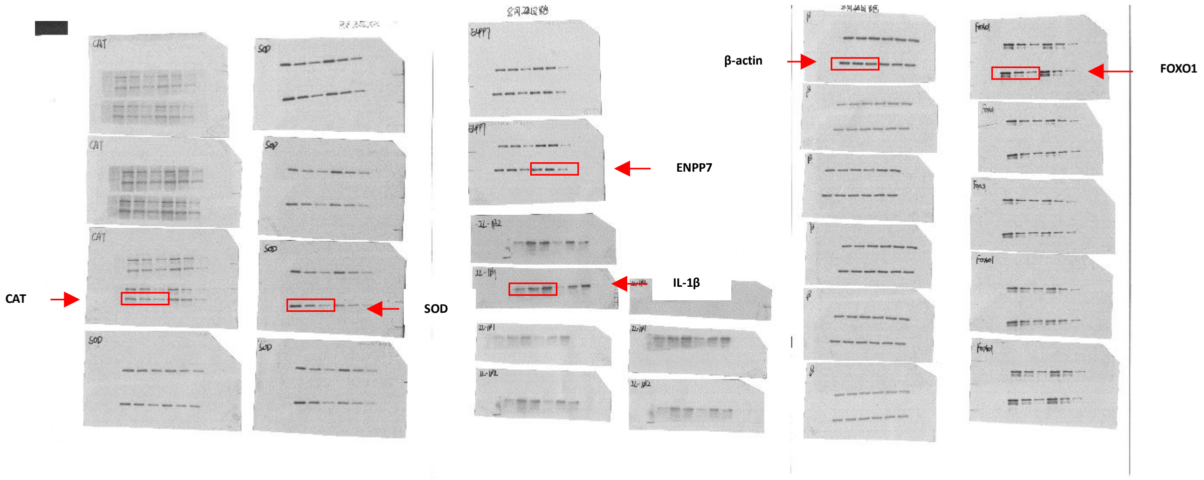

Each Western blot image submitted corresponds to experimental groups in the following loading order:

- **Lane 1 – NC (negative control)**
- **Lane 2 – NC + DSS**
- **Lane 3 – siENPP7 + DSS**

Lane 4–6 contain repeated samples of Lane 1–3, respectively, loaded again for technical consistency. Representative bands were selected from these lanes for the assembly of composite images presented in Figure 4g (red rectangles indicate the specific regions that were cropped and presented in the main manuscript figures)**.**

This loading sequence was used consistently across all relevant figures in the manuscript. The NC group represents untreated control cells, the NC + DSS group was treated with 1% DSS to induce inflammation, and the siENPP7 + DSS group represents cells transfected with ENPP7-targeting siRNA followed by DSS treatment.

We confirm that all lanes shown in the figure were loaded in this order and were derived from the same membrane without rearrangement.

Figure 5g Raw Western Blot Image

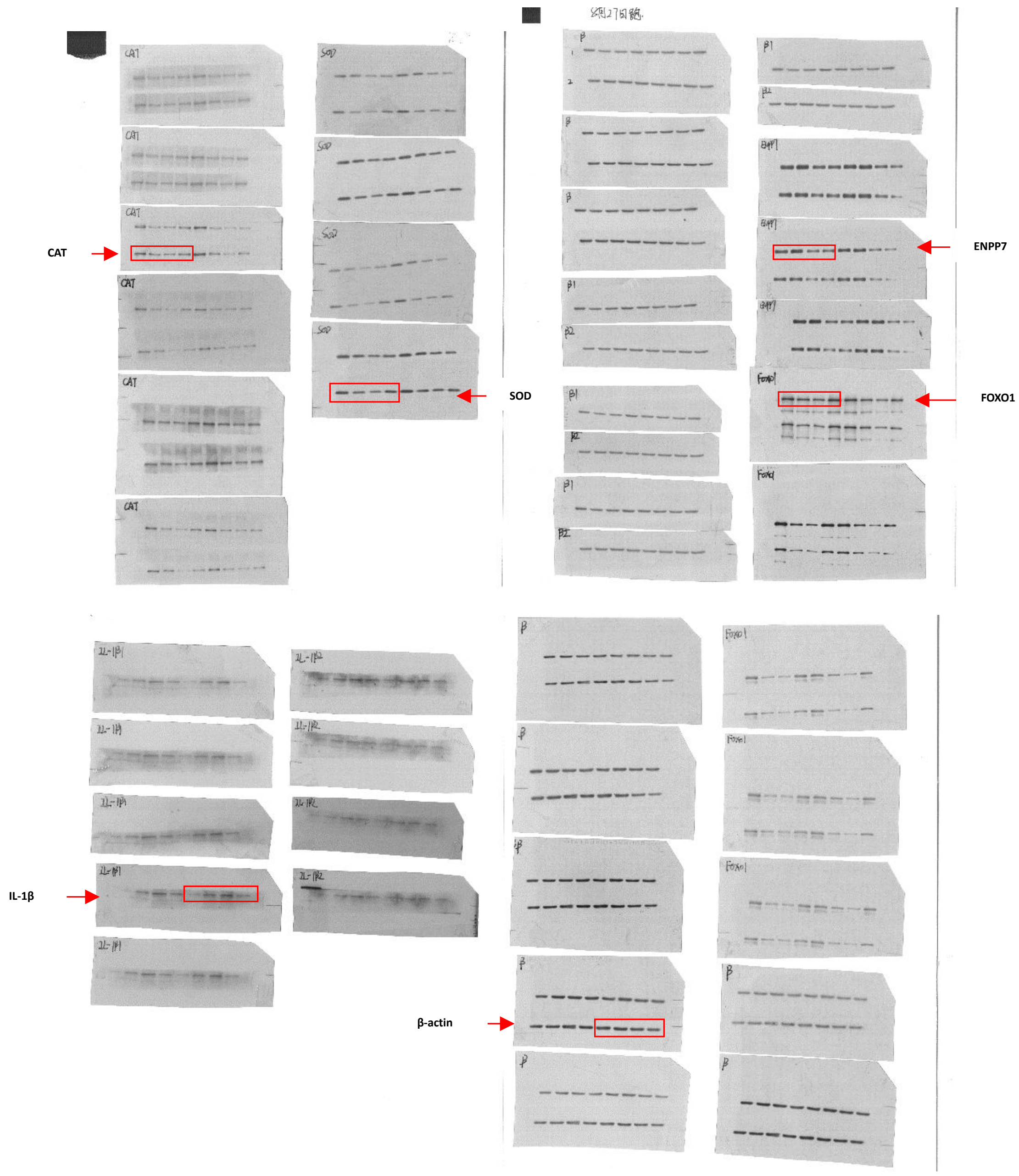

Each Western blot membrane corresponding to Figure 5g contains **eight sample lanes**. The **loading order** is as follows:

Lane 1 – NC (negative control); Lane 2 – NC + DSS; Lane 3 – siENPP7 + DSS; Lane 4 – siENPP7 + oeFOXO1 + DSS; Lanes 5–8 are technical repeats of **Lanes 1–4**, respectively, using the same set of samples loaded again to confirm reproducibility.

For the final figure assembly, we selected **representative bands** from the most consistent and clearly exposed lanes for presentation in **Figure 5g** (red rectangles indicate the specific regions that were cropped and presented in the main manuscript figures). These selected regions were cropped from the original full membrane, and **no image processing or enhancement was performed**. The bands used in the figure are clearly indicated in the raw data file.

Protein Molecular Weight Marker Information

- **Product Name:** PageRuler™ Prestained Protein Ladder, 10 to 180 kDa
- **Brand:** Thermo Scientific™
- **Catalog Number:** 26616
- **Molecular Weight Range:** 10–180 kDa
- **Band Colors:** Blue, orange, and green
- **Number of Bands:** 10
- **Application:** Suitable for SDS-PAGE and Western blotting. Used to monitor protein migration and transfer efficiency, and to estimate molecular weights of target proteins.
